# Cases of Tracheotomy and Diphtheria

**Published:** 1880-06

**Authors:** E. Fletcher Ingals

**Affiliations:** Lecturer on Physical Diagnosis and Diseases of the Chest and on Laryngology in Post Graduate Course, Rush Medical College; 188 Clark St., Chicago


### ARTICLE III.

CASES OF TRACHEOTOMY FOR CROUP AND DIPHTHERIA. By E. FLETCHER INGALS, A.M., M.D., Lecturer on Physical Diagnosis and Diseases of the Chest and on Laryngology in Post Graduate Course, Rush Medical College.

CASE I. About the middle of March, 1880, I was called by Dr. David Dodge to operate on —— Miles, a boy about six years of age, suffering from diphtheritic croup.

We found the child breathing laboriously and much exhausted, but not markedly cyanotic. Assisted by Drs. Dodge, Mulfinger and Waters, I operated at once.

Shortly after the introduction of the tube the child ceased breathing and was apparently dead, but the heart was found to be still beating, and the tube was clear. Artificial respiration and vigorous rubbing of the limbs, kept up for about twenty minutes, had the happy effect of restoring the patient. Subsequently the child did well. Lime water was kept boiling in the room and an atomizer was kept in use part of the time, throwing a spray of carbolic acid. The patient was kept on tonics, quinia and iron, and made a good recovery.

The tube was removed on the twelfth or thirteenth day (I am not quite certain which).

CASE II. —— Freer, æt. about two years, true croup. April 6, 1880. I saw the patient first about 9 a. m. on this date and found him breathing laboriously and partially cyanotic. I at once told the mother that an operation offered the only chance for saving her child. She wished it performed and I hastend for my instruments.

I returned about ten o'clock with Dr. D. W. Graham, who had kindly consented to assist me, and, finding the child unconscious and extremely cyanotic, we made preparations for the operation with the greatest despatch. When the child was placed on the table it manifested no sensation on touching the eyeball, therefore no anæsthetic was used. The operation was completed without difficulty, but, as I had at hand no tube of proper size, silk threads were passed through the trachea near the edges of the opening on each side and fastened with an elastic behind the neck. Two students, Messrs. T. E. Webb and L. W. Pontius, volunteered to stay with the patient and give all necessary care. The patient rallied and seemed very bright that evening, but early the next morning Mr. Pontius telephoned to me that the child was rapidly sinking. I hastened to the house and found the patient, as he had stated, in a precarious condition. The tissues about the wound were dry and the opening into the trachea partially contracted and the trachea itself partially filled with inspissated mucus.

I made a solution of bi-carbonate of soda, a teaspoonful to a pint of water, of which I directed a spray with the steam atomizer on the tracheal opening for about half an hour, by which time the mucus was softened, so that we removed it and, upon inserting a tube, the child again breathed easily and rapidly improved. In the evening the child continued better than early in the morning, but it showed some symptoms of filling of the bronchial tube with the croupy exudation.

Mr. P. remained with the child until one o'clock of that night, when it died from the gradually increasing obstruction below the trachea, thirty-nine hours after the operation. This patient had been hoarse for two or three days but had no alarming symptoms until the night previous to the operation. The mother had treated these as she had been accustomed to treat croup, by free use of substances which caused vomiting. After the operation the child was given fluid diet, which it took sparingly, and whisky, which was administered freely.

Case III. True Croup. April 11, 1880. L. Gingsburg, æt. six, had been sick about two days.

This case was very similar to the above. I was called by telephone at midnight to see "a child dying in croup," therefore I took my instruments with me. I found the child cyanotic, restless, breathing laboriously and nearly unconscious.

An operation was advised and consented to. I sent for Dr. Bartlett, who lived only a block distant, and, as soon as lamps could be procured, we made the operation. When the child was placed on the table she was nearly insensible but a small amount of chloroform was administered to keep her quiet.

The operation was made as the preceding and ligatures were passed for convenience through the cut edges of the trachea. This case progressed almost exactly as the preceding, and death occured in forty hours, from a similar cause. The treatment was the same as in the preceding, with the addition of the internal use of benzoate of soda. Mr. Pontius, who watched the preceding case for me, stood faithfully by the little patient to the last, and to his careful and assiduous attendance are to be attributed the last twenty hours of the patient's life.

The first of these cases was diagnosticated diphtheritic croup. It was operated on comparatively early and recovery occured.

The two latter were undoubtedly cases of true croup; they were operated on after they were moribund and their lives were prolonged about forty hours, to the great comfort of parents and friends, and death finally came quietly, without a struggle.

188 Clark St., Chicago.

